# Doctor, When Should I Start Walking? Revisiting Postoperative Rehabilitation and Weight-Bearing Protocols in Operatively Treated Acetabular Fractures: A Systematic Review and Meta-Analysis

**DOI:** 10.3390/jcm13123570

**Published:** 2024-06-18

**Authors:** Vincenzo Giordano, Robinson Esteves Pires, Luiz Paulo Giorgetta de Faria, Igor Temtemples, Tomas Macagno, Anderson Freitas, Alexander Joeris, Peter V. Giannoudis

**Affiliations:** 1 Serviço de Ortopedia e Traumatologia Prof. Nova Monteiro, Hospital Municipal Miguel Couto, Rio de Janeiro 22430-160, Brazil; lpgiorgetta@gmail.com (L.P.G.d.F.); igortemtemples@hotmail.com (I.T.); 2 Departamento do Aparelho Locomotor, Escola de Medicina, Universidade Federal de Minas Gerais, Belo Horizonte 30130-100, Brazil; robinsonestevespires@gmail.com; 3 Hospital Sirio Libanés, Buenos Aires C1419, Argentina; tomimacagno@gmail.com; 4 HOME—Hospital Ortopédico e Medicina Especializada, Brasília 70200-730, Brazil; andfreitas28@gmail.com; 5 Clinical Science, AO Innovation Translation Center, 8600 Dübendorf, Switzerland; alexander.joeris@aofoundation.org; 6 Department of Trauma & Orthopaedic Surgery, School of Medicine, University of Leeds, Leeds LS2 9LU, UK; pgiannoudi@aol.com

**Keywords:** acetabular fracture, weight-bearing, rehabilitation, PROMs, quality of reduction

## Abstract

**Background and Objectives**: Management of acetabular fractures is aimed at anatomically reducing and fixing all displaced or unstable fractures, as the accuracy of fracture reduction has been demonstrated to strongly correlate with clinical outcomes. However, there is a noticeable gap in the literature concerning the perioperative and postoperative care of patients with acetabular fractures, which ultimately can be potential risk factors for adverse outcomes and permanent disabilities. This study aimed to systematically review the available literature regarding rehabilitation practices, including weight-bearing protocols, across time points in surgically treated acetabular fracture patients and correlate these practices with functional outcomes. **Methods**: We systematically reviewed the Medline and PubMed databases and the Cochrane Central Register of Controlled Trials in accordance with Preferred Reporting Items for Systematic Reviews and Meta-Analyses (PRISMA) guidelines. The inclusion criteria were studies with adult patients (19+ years), publications from the last 10 years, articles focusing on rehabilitation or mentioning any aspect related to rehabilitation (such as weight-bearing or muscle training), and describing the surgical management of acute, isolated acetabulum fractures. Specific information was collected, including the fracture classification, time to surgery, surgical approach, surgical time, blood loss, fixation strategy, quality of reduction, postoperative rehabilitation protocol, complication rate, type(s) of complication, and outcome measurement(s). The choice(s) of surgical approach, surgical time, blood loss, and fixation strategy were stratified based on the fracture classification. The complication rate and type(s) of complication were calculated for all studies. Fractures were classified based on the Letournel classification. **Results**: A total of 494 articles were identified from the initial search, of which 22 (1025 patients) were included in the final review. The most common rehabilitation protocol favored isometric quadriceps and abductor strengthening exercises starting on the first postoperative day, with passive hip movement at 1–3 days postoperatively and active hip movement ranging from the first postoperative day to 4 weeks postoperatively. Partial weight-bearing with a walker or a pair of crutches was permitted from 1 to 12 weeks after surgery, and full weight-bearing was allowed depending on the patient’s general condition and fracture healing state (generally at the end of 3 months). In only three studies did the patients start bearing weight in the early postoperative period (≤1 week). Meta-regression analysis was not performed due to the discrepancy between studies that reported a weight-bearing protocol ≤1 week and >1 week postoperatively. **Conclusions**: Our study suggests that an accelerated postoperative rehabilitation protocol, including early permissive weight-bearing, does not appear to increase the risk of loss of reduction or the rate of complications after surgical treatment of acetabular fractures. However, a proper meta-analysis was not possible, and the heterogeneity of the included studies did not allow us to conclude anything about the potential biomechanical and clinical benefits nor the negative effects related to this rehabilitation regimen in terms of functional results. There is an inconsistent use of PROMs for objectively calculating the effect size of the accelerated protocol compared with restricted weight-bearing regimes. We pose the need for higher-level evidence to proof our hypothesis.

## 1. Introduction

The management of acetabular fractures is aimed at anatomically reducing and fixing all displaced or unstable fractures, as the accuracy of the fracture reduction has been demonstrated to strongly correlate with clinical outcomes [1,2]. Following these fundamental principles, historically good-to-excellent functional outcomes have been reported in up to 80% of operatively treated patients [1,3,4]. However, it has been pointed out that overall, studies lack high-quality data on medium- and long-term functional follow-ups, which can theoretically bias the existing reported outcome [5]. In addition, notable changes in several aspects of acetabular fractures have been occurring, as patients are getting older and sustaining different spectra of fracture patterns [5].

At present, understanding the fracture classification, performing a proper exposure, achieving an anatomic reduction, understanding screw trajectories, and assessing intra- and postoperative reduction are still the mainstays of acetabular fracture surgical management [2,5]. While considerable attention has been dedicated to enhancing surgical skills and techniques, as well as identifying potential risk factors for adverse outcomes and permanent disabilities, there remains a noticeable gap in the literature concerning the perioperative and postoperative care of patients with acetabular fractures.

In this scenario, evaluating the effect of modifiers on perioperative and postoperative care, such as rehabilitation protocols and programs, seems to be essential to have a coherent sense of their impact on surgical outcomes. Rehabilitation of operatively treated orthopedic trauma patients has been demonstrated to be an integral part of the management, providing optimal care to injured patients [6,7]. Specifically, in elderly patients with hip fractures, proper rehabilitation after surgery has been shown to shorten hospital stays, improve physical function, help patients maintain independent daily lives, and reduce the burden of medical treatment [8,9,10].

Our hypothesis is that standardized protocols for rehabilitation and weight-bearing restrictions following operative treatment of acetabular fractures are scarce in the literature. This gap in standardized protocols poses a challenge for orthopedic trauma surgeons and physical therapists aiming to optimize patient outcomes and ensure consistent quality of care. Addressing this issue is crucial for developing evidence-based guidelines for conducting rehabilitation efforts and weight-bearing protocols, ultimately improving the overall management of acetabular fractures and enhancing patient recovery. The aim of this systematic review and meta-analysis is to identify previously described rehabilitation practices, including weight-bearing protocols, across time points in surgically treated acetabular fracture patients and correlate these practices with functional outcomes.

## 2. Materials and Methods

This study was designed and conducted according to the guidelines proposed by the Cochrane Handbook for Systematic Reviews of Interventions (http://handbook-5-1.cochrane.org, accessed on 2 January 2024), and it was reported in compliance with the Preferred Reporting Items for Systematic Reviews and Meta-Analyses (PRISMA) statement guidelines [11]. As this study was based on previously published studies, ethical approval and patient consent were not needed.

### 2.1. Search Strategy

**Figure 1:**
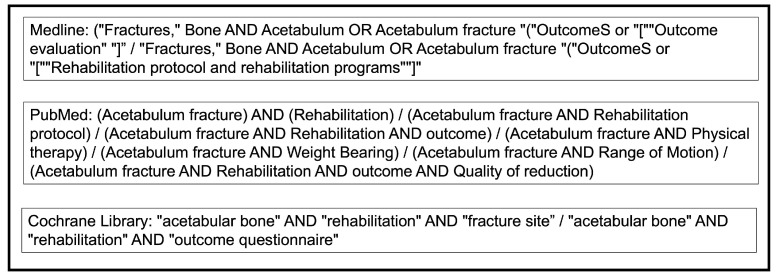
Systematic review search strategy.

A systematic review was performed using Medline and PubMed databases, as well as the Cochrane Central Register of Controlled Trials between August and December 2023, focusing on acetabular fracture rehabilitation and treatment outcomes. Searches were limited to human studies and the English language. The terms acetabulum fracture and rehabilitation were explored to obtain related search terms and categories using database-appropriate search terms (Figure 1).

The inclusion criteria were adult patients (19+ years), publications from the last 10 years, articles focusing on rehabilitation or mentioning any aspect related to rehabilitation (such as weight-bearing or muscle training), and describing the surgical management of acute, isolated acetabulum fractures. The exclusion criteria were pathological fractures, acetabular fractures treated primarily with joint replacement, failure to inform about the follow-up period, nonexistence of evaluation criteria, experimental studies involving animals, cadaveric specimens, or biomechanical testing, review articles, case reports and small case series (<10 cases), expert opinions, and letters to the editor.

### 2.2. Study Selection

Based on the titles and abstracts, three investigators (V.G., L.P.G.d.F., and T.M.) picked out the potential eligible studies. All duplicate titles were removed. Then, the full text of the remaining studies was reviewed for eligibility by the same three investigators, and any disagreements were resolved by discussions involving the other authors for the final decision. All studies were independently assessed to check whether they met the inclusion criteria. Again, if there was a disagreement regarding inclusion, it was resolved by a discussion involving the other authors for the final decision, and if no consensus could be reached, then the study was excluded.

### 2.3. Data Extraction and Outcome Measures

After full-text articles were selected, two investigators (V.G. and L.P.G.d.F.) independently performed data extraction and evaluated the quality of the included studies using the Methodological Index for Non-Randomized Studies (MINORS) criteria, a valid instrument designed to assess the methodological quality of non-randomized surgical studies, whether comparative or non-comparative [12]. Basic information was collected, including the journal name, author(s), year published, level of evidence, number of fractures, and follow-ups. Specific information was collected, including the fracture classification, time to surgery, surgical approach, surgical time, blood loss, fixation strategy, quality of reduction, postoperative rehabilitation protocol, complication rate, type of complication(s), and outcome measurement(s). The choice of surgical approach(es), surgical time, blood loss, and fixation strategy were stratified based on the fracture classification. The complication rate and type(s) of complication were calculated for all studies. Fractures were classified based on the Letournel classification [13,14].

A full narrative synthesis of the included studies was conducted, focusing on correlating the outcome measurements with the postoperative weight-bearing regimen. When possible, quantitative synthesis was undertaken for the postoperative outcome measurements to compare between non-weight-bearing and weight-bearing postoperative regimens and determine the difference in functional outcomes between groups. In cases with no comparison between groups in terms of weight-bearing regimens, single-arm meta-analysis was undertaken to pool the results of the single-arm observational studies and assess the overall outcome measurement. All relevant information from each article was extracted and inserted into an Excel document.

### 2.4. Assessment of the Risk of Bias

Initially, each primary study was assessed based on its OCEBM level of evidence (LoE) [15]. The potential presence of publication bias was first explored visually by generating the respective funnel plots for the main outcomes of interest. A symmetrical distribution of the studies about the pooled effect estimate would be interpreted as an absence of publication bias. Furthermore, we utilised Egger’s test and Begg’s rank test. For both tests, there is an indication of publication bias when the two-sided *p* value is extremely low (below the significance level). Sensitivity analysis was performed by repeating the pooling process after eliminating studies of either a low methodological rating with the MINORS criteria or dubious eligibility [12]. Should this process not yield considerably different results than the ones originally obtained, our confidence surrounding the robustness of our findings would increase. The risk of bias was assessed using the Cochrane robvis visualization tool and evaluated according to the Risk of Bias in Non-randomized Studies of Interventions (ROBINS-I) tool for studies that were not randomized [16].

### 2.5. Statistical Analysis

Data were extracted and entered into an Excel spreadsheet. Statistical analysis was carried out to estimate the overall event rate using Comprehensive Meta-Analysis software (version 2.0; Biostat, Inc., Englewood, NJ, USA). The Yates-corrected chi-squared test was used to assess categorical variables, and the Kruskal–Wallis test was used for independent measures. After this, the Shapiro–Wilk test was used preliminarily to test the normality and homogeneity of the variables assessed. A *p* value <0.05 was considered significant.

## 3. Results

### 3.1. Selection of Studies

**Figure 2:**
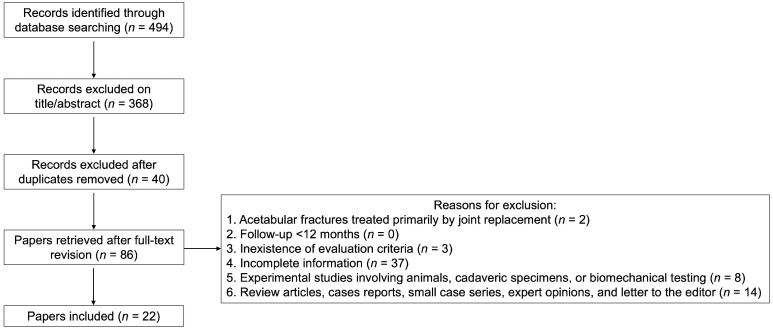
Preferred Reporting Items for Systematic Reviews and Meta-Analyses (PRISMA) flow diagram displaying number of studies retrieved following searches and removal criterion at each screening stage.

A total of 494 records were identified through database searches. After screening titles and abstracts and removing duplicates, 86 potentially relevant records met our eligibility criteria, and full-text articles were selected. Of these, 64 records were discarded, leaving 22 records (1025 patients) that were included in the final review. The search strategy for databases with a flowchart of the literature selection process is outlined in Figure 2.

### 3.2. Characteristics of the Studies

**Figure 3:**
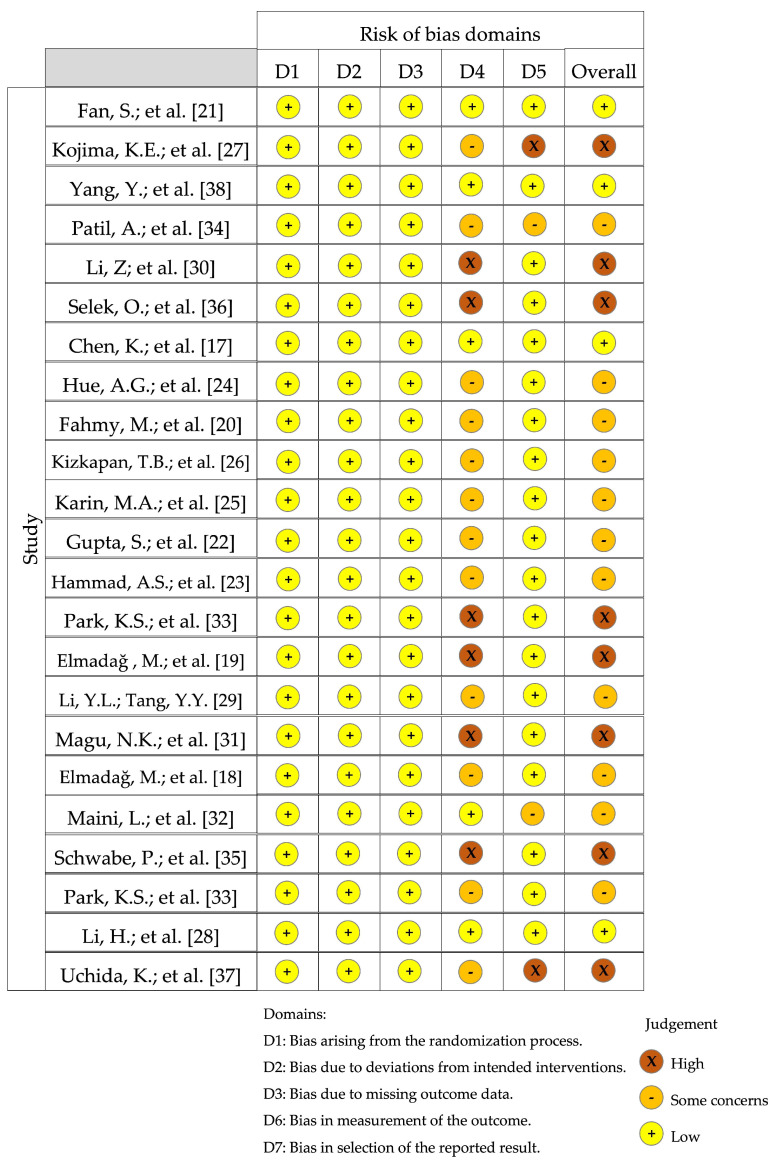
Results of the Risk of Bias in Non-randomized Studies of Interventions (ROBINS-I) tool for non-randomized studies, visualized in traffic light plots for each individual domain assessed using Cochrane robvis visualization tool [17,18,19,20,21,22,23,24,25,26,27,28,29,30,31,32,33,34,35,36,37,38].

All studies included in the final analysis were either individual cohort studies (*n* = 3), representing a grade of recommendation of B and level of evidence of 2b, or low-quality cohort or case-control studies (*n* = 19), representing a grade of recommendation of C and level of evidence of four [17,18,19,20,21,22,23,24,25,26,27,28,29,30,31,32,33,34,35,36,37,38]. All studies were assessed using the MINORS criteria, which contain 12 items, with the first eight being specifically for non-comparative studies, giving a maximum score of 16 for non-comparative studies and 24 for comparative studies. The maximum score indicates that methodological items were adequately reported. The mean score was 12.8 (range: 9–15) for non-comparative studies and 19.5 (range: 18–21) for comparative studies. Unbiased assessment of the study’s endpoint and lack of prospective calculation of the study size were the main problems encountered in the non-comparative studies, whereas unbiased assessment of the study’s endpoint, lack of prospective calculation of the study size, and inadequate statistical analyses were the main problems of the comparative studies. Most studies were published in 2014 (*n* = 6), 2017 (*n* = 4), and 2018 (*n* = 4). Six studies were published in *Injury* (Elsevier Ltd. (Amsterdam, The Netherlands); 2022 Journal Impact Factor: 2.9; 2022 Journal Citation Indicator (JCI): 0.850) [20,23,25,28,29,33], and two studies each were published in the *Journal of Orthopaedic Trauma* (Wolters Kluwer Health, Inc. (Baltimore, MD, USA); 2022 Journal Impact Factor: 2.3) [17,35], *Orthopaedic Surgery* (Tianjin Hospital and John Wiley & Sons Australia, Ltd. (Chicago, IL, USA); 2022 Journal Impact Factor: 2.1; 2022 Journal Citation Indicator (JCI): 0.85) [21,38], and *Orthopaedic Traumatology Surgery and Research* (BioMed Central Ltd. (London, UK); 2022 Journal Impact Factor 2.6; 2022 SCImago Journal Rank (SJR): 0.744) [18,24]. The characteristics of the included studies are presented in Table 1. The risk of bias is shown in Figure 3 [16].

### 3.3. Sample Demographics

The minimum number of patients included in a study was 12, and the maximum was 178, with a mean of 46.6 and a median of 32 (95% CI: 44.2–49.0). Of the 22 studies included, in 20 studies (929 patients), it was possible to establish a male-female relationship, with 721 (77.6%) men and 207 (22.3%) women (*p* = 0.0005) [17,18,19,20,21,23,24,25,26,27,28,29,30,31,32,33,34,35,36,38]. One study (25 patients) did not present the male-female ratio [22], and in another (91 patients, 71 of whom underwent open reduction and internal fixation (ORIF) of the fracture), the male-female ratio was not established for the patients treated surgically [37]. The average age ± standard deviation (SD) of patients was 42.8 ± 10.9 years. In a study by Uchida et al. [37] including 21 patients treated non-surgically and 71 patients treated with ORIF, there was no description for each of the groups, with a patient general mean age of 49.0 years old (range: 18–80 years old).

All studies classified the fractures using the Letournel classification [13,14]. There were 401 elementary fractures (82 anterior column fractures, 14 anterior wall fractures, 25 posterior column fractures, 210 posterior wall fractures, and 70 transverse fractures) and 614 associated fractures (42 posterior column posterior wall fractures, 104 transverse posterior wall fractures, 114 anterior column posterior hemitransverse fractures, 99 T-type fractures, and 255 both-column fractures). There was no significant difference between the number of elementary and associated acetabular fractures (*p* = 0.21). Three fractures were considered non-classifiable in a study by Kojima et al. [27], and one fracture was classified as an isolated quadrilateral plate in a study by Li and Tang [29]. Twelve studies mentioned the occurrence of 236 associated skeletal injuries, 32 non-skeletal (excluding neurological) injuries, 11 peripheral nerve injuries, and 8 non-specified multiple traumas [18,20,21,24,25,26,27,28,30,31,35,38]. Although reported more, there was no statistical significance between skeletal injuries and non-skeletal injuries, including peripheral nerve injuries (*p* = 0.09). Also, there was no significant difference between non-skeletal injuries and peripheral nerve injuries (*p* > 0.05). The demographics are outlined in Table 2.

### 3.4. Perioperative Parameters and Form of Treatment

The mean time to surgery was 6.4 ± 2.7, ranging from 1 day to 32 days [17,18,20,21,22,23,24,25,26,28,29,32,33,34,37,38]. This was reported in 11 studies as a mean and range, as a mean alone in four studies, and as an interval of time in one study. Six studies did not mention this information. Twenty studies mentioned the type of surgical approach they used [17,18,19,20,21,22,23,24,25,26,28,29,30,31,32,33,34,35,37,38], whereas in two studies, these data were not available. The most frequently used approach was the Kocher–Langenbeck approach in 351 cases (34.2%), either isolated (*n* = 231), associated with digastric trochanteric flip osteotomy (*n* = 50), or combined with other approaches (*n* = 70), followed by the ilioinguinal approach in 128 cases (12.5%). Other approaches, including percutaneous fixations, were less commonly used. The mean blood loss was reported in 10 studies, ranging from 150 mL to 2150 mL [17,18,19,20,21,25,28,30,32,38]. This was reported as a mean and range in seven studies, one study presented the range alone, and two studies presented the mean alone. The mean surgical time was reported in nine studies, ranging from 35 min to 360 min [17,21,24,25,28,30,32,33,38]. This was reported as a mean and range in six studies and as the range alone in three studies. In only five studies, details for the surgical approach, mean blood loss, and mean surgical time were given for a total of 303 (37.6%) patients [17,21,28,32,38]. In this subgroup of studies, the Kocher–Langenbeck approach was used in 103 cases, the ilioinguinal approach was used in 22 cases, the lateral-rectus approach was used in 178 cases, and in 48 cases, this information was not available. The mean blood loss for the patients operated on through the Kocher–Langenbeck approach was 483.8 ± 232.9 mL, with a mean surgical time of 135.3 ± 12.2 min [28,32,38]. The mean blood loss for the patients operated on through the ilioinguinal approach was 793 ± 228 mL, with a mean surgical time of 182 ± 40 min [17]. The mean blood loss for the patients operated on through the lateral-rectus approach was 440 ± 153 mL, with a mean surgical time of 75 ± 29 min [21]. Due to the small sample size, no correlation was possible between the surgical approach, mean blood loss, and mean surgical time. Seventeen studies mentioned the type of implant used for fracture fixation, which varied from conventional non-locked implants (3.5 mm reconstruction plates, one-third tubular plates, small fragment cortical and cancellous screws, large fragment cancellous screws, cables, and 7.3 cannulated screws) to locked implants (3.5 mm reconstruction plates and screws and precontoured anatomical plates) [17,19,20,21,22,23,24,25,26,28,29,30,31,32,33,35,36,38]. The perioperative parameters and form of treatment of the included studies are presented in Table 3.

### 3.5. Quality of Reduction

The quality of fracture reduction was reported in 20 studies, with 964 fractures, 59 anterior column fractures, 14 anterior wall fractures, 19 posterior column fractures, 195 posterior wall fractures, 65 transverse fractures, 42 posterior column posterior wall fractures, 100 transverse posterior wall fractures, 106 anterior column posterior hemitransverse fractures, 99 T-type fractures, 255 both-column fractures, 1 isolated quadrilateral plate fracture, and 3 non-classified fractures [17,19,20,21,23,24,25,26,27,28,29,30,31,32,33,34,35,36,37,38]. In 12 studies, the quality of reduction was classified according to the Matta scoring system, in which 1.0 mm or less of displacement or step-off is rated as an anatomic or excellent reduction; 2.0–3.0 mm of displacement or step-off is rated as imperfect, good, or satisfactory reduction; and more than 3.0 mm of displacement or step-off is rated as poor or unsatisfactory reduction [39,40]. In this subgroup of patients, the quality of reduction was considered anatomic or excellent in 347 (69.4%) cases, imperfect, good, or satisfactory in 111 (22.2%) cases, and poor or unsatisfactory in 42 (8.4%) cases [17,19,20,21,23,24,25,28,32,33,37,38]. A further six studies reported their results as “excellent”, “good”, “fair”, or “poor” [26,28,29,30,31,36]. In one study, the authors stated that results were “acceptable” [34], and in another study, the results were described as “satisfactory” or “unsatisfactory” [27].

### 3.6. Postoperative Rehabilitation Protocol

A range of postoperative rehabilitation protocols was described in the 22 included studies according to timing, range of motion, muscle training, and weight-bearing [17,18,19,20,21,22,23,24,25,26,27,28,29,30,31,32,33,34,35,36,37,38]. Overall, isometric quadriceps- and abductor-strengthening exercises were started on the first postoperative day, with passive hip movement at 1–3 days postoperatively and active hip movement ranging from the first postoperative day to 4 weeks postoperatively. Partial weight-bearing with a walker or a pair of crutches was permitted from 1 to 12 weeks after surgery, and full weight-bearing was allowed depending on the patient’s general condition and fracture healing state, generally at the end of 3 months. A faster weight-bearing protocol was performed by Schwabe et al. [35], who utilized a supervised mobilization regimen with 30 kg of weight-bearing on the ipsilateral extremity with crutches or a mobile walking device, which started on the first day after the operation, and full weight-bearing after 6 weeks postoperatively. On the contrary, more prolonged weight-bearing protocols were described by Kizkapan et al. [26] and Maini et al. [32], with patients being allowed to perform partial weight-bearing 3 months after surgery and start full weight-bearing between 4 and 6 months after surgery.

Postoperative skeletal traction was described in three studies [20,32,38]. Fahmy et al. [20] kept their patients on absolute bed rest with continuous transtibial traction for 6 weeks and started passive mobilization of the operated hip after the 10th postoperative day. Maini et al. [32] used skeletal traction for 3 weeks followed by a non-weight bearing status for 6–12 weeks, depending on the stability and fixation of the joint. Finally, Yang et al. [38] maintained skeletal traction for 2–4 weeks in patients with traumatic posterior hip dislocation before starting functional hip exercise.

### 3.7. Outcome Measurements and Complications

The outcome measurements were described in 19 studies [17,18,19,20,21,22,23,25,26,28,29,30,31,32,33,34,35,36,38]. The authors used the modified Merle D’Aubigné and Postel score (MDPS) in 16 studies [17,18,19,20,21,22,23,25,26,28,30,31,33,34,35,36,38] and the Harris hip score (HHS) in six studies [18,19,29,33,34,35]. In four studies, both the MDPS and the HHS were used [18,19,34,35], but Patil et al. [34] reported their results as means only (modified MDPS of 14.95 ± 3.46 and HHS of 85.48 ± 2.97). In one study, although the functional outcome was reported, no recognized outcome evaluation score was mentioned [32].

Overall, in 15 studies, the MDPS was considered excellent to good in 626 (83.8%) patients and fair to poor in 121 (16.2%) patients [17,18,19,20,21,23,25,26,28,30,31,32,34,35,36,38]. In a study by Gupta et al. [22] with 25 displaced acetabular fractures treated by ORIF, outcome scoring was presented for 24 patients only, and thus it was not considered for sample calculations. Regarding the HHS, overall, the outcomes were considered excellent to good in 134 (56.5%) cases and fair to poor in 103 (43.5%) cases.

Twenty-one studies commented on complications, ranging from “no complications” to common non-fatal complications associated with acetabular fracture management [17,18,19,20,21,22,23,24,25,26,27,28,29,30,31,32,33,34,35,37,38]. Heterotopic ossification was the most reported skeletal complication, occurring in 52 (5.1%) cases, followed by posttraumatic hip arthritis in 41 (4.0%) cases and avascular necrosis (AVN) of the femoral head in 17 (1.6%) cases. Thromboembolic complications were reported in 24 (2.3%) cases, namely 24 lower extremity deep venous thrombosis (DVT) and symptomatic pulmonary embolism cases, which were treated with anti-coagulation. Postoperative peripheral nerve injuries were seen in 28 (2.7%) cases, with 14 involving sciatic nerve palsy, 13 involving lateral femoral cutaneous nerve palsy, and 1 case of obturator nerve palsy. Other complications were reported less frequently. Schwabe et al. [35] operated on 12 patients with percutaneous reduction and fixation with no reported complications.

Follow-ups ranged from 6 weeks to 9 years. The quality of reduction, postoperative rehabilitation protocol, and outcome measurements of the included studies are presented in Table 4. The follow-up periods and complications are outlined in Table 5.

### 3.8. Meta-Analytic Regression

**Figure 4:**
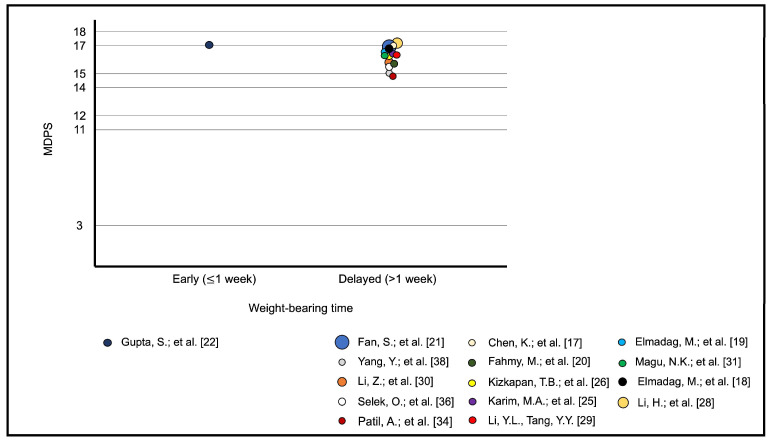
Bubble plot of functional outcomes of the mean MDPS versus early (≤1 week) and delayed (>1 week) weight-bearing times [17,18,19,20,21,22,25,26,28,29,30,31,34,36,38].

**Figure 5:**
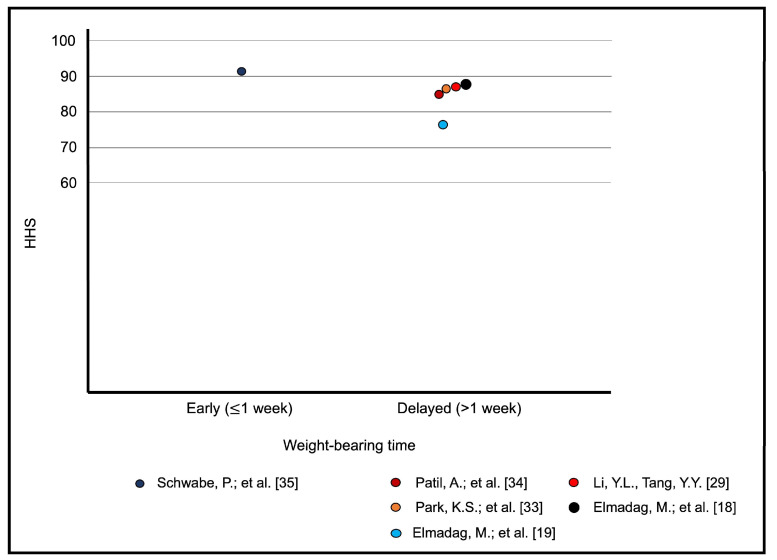
Bubble plot of functional outcomes of the mean HHS versus early (≤1 week) and delayed (>1 week) weight-bearing times [18,19,29,33,34,35].

The only study to assess non-weight-bearing versus weight-bearing regimens did not report any outcome evaluation score. In a therapeutic retrospective cohort study with 137 patients with displaced acetabular fractures that underwent ORIF, it was observed that loss of reduction (increase of >2 mm in either the articular step or gap) due to early weight-bearing presented a relative risk of 1.506 (CI: 0.503–4.514), with no significant difference between the two groups (*p* = 0.664) [27]. In two other studies, patients started weight-bearing in the early postoperative period (≤1 week). In a retrospective case series with 22 consecutive patients submitted to three-dimensional fluoroscopy-navigated percutaneous screw fixation of minimally displaced acetabular fractures, there was no complication, with excellent results in eight patients and good results in four patients based on the HHS system [35]. All patients underwent supervised mobilization with 30 kg of weight-bearing on the ipsilateral extremity with crutches or a mobile walking device starting on the first postoperative day, and full weight-bearing was permitted after 6 weeks postoperatively. In a retrospective study of prospectively collected data from 25 patients with displaced acetabular fractures treated by ORIF using the Kocher–Langenbeck approach along with trochanteric flip osteotomy, excellent-to-good results were observed in 22 (88.0%) cases [22]. All patients were allowed to perform toe-touch weight-bearing within the first week, and full weight-bearing was permitted at the end of 3 months. There were three complications, none of which were related to the weight-bearing regimen. In the remaining studies, partial weight-bearing was permitted after at least 4 weeks after surgery, showing high overall heterogeneity. A bubble plot of the functional outcome (mean MDPS and mean HHS) versus weight-bearing time (early (≤1 week) and delayed (>1 week)) was established (Figure 4 and Figure 5), but meta-regression analysis was not performed due to the discrepancy between studies that reported a weight-bearing protocol ≤1 week and >1 week postoperatively. Because some of the included studies did not report the mean scores, the mean values within the MDPS (excellent = 18, good = 16, fair = 13, and poor = 7 points) and the HHS (excellent = 95, good = 85. regular = 75 and poor = 35 points) were adopted for the purpose of calculating the averages. For this calculation, the study by Hammad et al. was excluded [23].

## 4. Discussion

Our study demonstrated scarce evidence when comparing the relationship between the postoperative rehabilitation protocols, especially weight-bearing, and the most commonly used patient-reported outcome measure scores for patients treated surgically for acetabular fractures. Although the outcome measures were described in 19 of the 22 included studies [17,18,19,20,21,22,23,25,26,28,29,30,31,32,33,34,35,36,38], meta-regression analysis was not possible due to discrepancies between studies, especially in relation to weight-bearing protocols ≤1 week and >1 week postoperatively. Of the 15 studies in which the authors used the modified Merle D’Aubigné and Postel score included in the meta-regression analysis, in only one were patients encouraged to perform an early weight-bearing protocol [17,18,19,20,21,22,25,26,28,30,31,34,35,36,38]. The same was observed in the six studies in which the authors used the Harris hip score, where only one described an early weight-bearing protocol [18,19,29,33,34,35]. The reason why restricted or limited delayed weight-bearing after acetabular fracture fixation is preferred among surgeons is unclear, as most orthopedic surgeons routinely prescribe at least partial weight-bearing for a lower extremity fracture in an attempt to produce an optimal mechanical environment at various stages of fracture healing [41]. From a biomechanical point of view, weight-bearing produces micromovements between the fracture fragments, stimulating bone consolidation, which clinically implies rapid functional recovery and a faster return to activities of daily living, thus potentially reducing complications associated with late rehabilitation protocols [42,43].

The management of acetabular fractures should be aimed at anatomical reduction and stable fixation of all displaced or unstable fractures, thus enabling early patient rehabilitation [2]. However, the literature on weight-bearing status after fixation of an acetabulum fractures continues to empirically suggest at least 6 weeks of non-weight-bearing as the most prevalent postoperative treatment option, mainly due to fear of implant failure, fracture displacement, and loss of reduction. This more conservative recommendation appears to be motivated by the belief that by reducing the forces acting at the fracture site, the bone and ligament healing processes will occur without greater risk of fixation failure [42,43,44]. In fact, in the 22 studies included [17,18,19,20,21,22,23,24,25,26,27,28,29,30,31,32,33,34,35,36,37,38], we consistently observed that active and passive hip movements and isometric quadriceps- and abductor-strengthening exercises were started between one and three days postoperatively, and weight bearing was basically postponed depending on the patient’s general condition and fracture healing state. Interestingly, non-weight bearing has been shown to be associated with greater energy expenditure and higher contact pressures at the hip joint, with peak pressures in the acetabulum during sitting and standing activities performed during restricted weight-bearing being nearly three times the forces observed during walking [42,43,45,46]. Moreover, much of the force during sitting and standing activities is directed posteriorly, whereas during ambulation, more load is distributed to the upper lateral surface of the roof [42,45,46].

Although it is well recognized that the quality of reduction remains the most important factor for a better functional outcome, other factors have been identified as also affecting the functional recovery of the hip after an acetabular fracture, including the patient’s involvement in the rehabilitation protocol [47]. In this scenario, addressing the issue of rehabilitation after surgical treatment of acetabular fractures gains importance, especially when some authors have not reported loss of reduction or an increase in complications when allowing early permissive partial weight-bearing, even after percutaneous fixations using screws only [22,27,35,43,48]. Kojima et al. [27] showed that different weight-bearing regimes (early vs. late) did not influence the quality of reduction in 137 patients with surgically treated displaced acetabular fractures. Schwabe et al. [35] found no complications in 22 consecutive patients submitted to percutaneous screw fixation of minimally displaced acetabular fractures, who utilized a supervised mobilization weight-bearing regimen which started on the first day after the operation and full weight-bearing after 6 weeks postoperatively. Similarly, Kazemi and Archdeacon [48] found no loss of reduction and observed similar results in 22 patients who underwent percutaneous fixation with screws and were allowed immediate full weight-bearing postoperatively. Not surprisingly, the benefits observed from faster recovery in the immediate postoperative period, including early permissive weight-bearing, have shown similar results and complications in other articular fractures of the lower limbs [49,50,51,52].

Another aspect that must be taken into consideration and that is constantly neglected in the description of the follow-up after fixation of an acetabulum fracture is the low adherence of patients to medical restrictions regarding weight-bearing in the postoperative period. Chiodo et al. [53] measured the compliance with a period of prescribed non-weight-bearing in 51 consecutive adult orthopedic patients with unilateral lower-extremity injuries or procedures. The noncompliance rate with the postoperative non-weight-bearing restriction was 27.5%, despite explicit instructions and education about possible complications associated with weight-bearing. No significant influence on weight-bearing performance was found regarding sex, age, language spoken, body mass index, time in a cast, or the treating surgeon. Braun et al. [42] continuously monitored 10 patients after surgical treatment of an acetabulum fracture with a pressure-measuring insole. In the postoperative protocol, the authors instructed every patient to maintain a 20 kg weight-bearing limit for 6 weeks, but only one patient stayed within the weight-bearing limit during the analysis. Despite almost all patients exceeding the prescribed amount of weight-bearing, the postoperative reduction was maintained at the 6 week timepoint, suggesting that higher plantar loading has no effect on the early radiographic results [42].

When bringing together all the pros and cons of reported rehabilitation protocols, it seems that immediate or early weight-bearing may not place patients at an excessive risk of displacement after fixation of an acetabulum fracture. Although the quality of reduction and fixation techniques remains the most important factor in obtaining satisfactory outcomes after surgical treatment of acetabular fractures, our study highlights the need to review current postoperative weight-bearing protocols. The ability to walk has been shown to be an important aspect in patients’ self-perception regarding activities of daily living, quality of life, and pain. Although the “ability to walk” is one of the items assessed in both MDPS and HHS, the expected benefit of early permissive weight-bearing was not assessed in high-quality, prospective, randomized controlled trials, and its effect size was not calculated for comparison with restricted weight-bearing regimens either. Therefore, the effectiveness of a faster postoperative protocol involving permissive weight-bearing, in addition to measures traditionally described in terms of analgesia, recovery of articular mobility, and muscle strengthening, should be tested, and the outcomes should be assessed and compared to the quality of reduction, complications, and quality of life. Other authors have proposed the same when finding no difference in functional outcome scores or complication rates between early and late weight-bearing protocols for surgically treated acetabular fractures [44,54]. Currently, several authors have studied the applicability of innovative gait rehabilitation protocols for temporary or permanent gait impairments, since poor balance control during one’s gait and postural maintenance have been associated with disabilities, falls, and increased mortality [55,56,57]. These concepts can be extended in the future to rehabilitate patients after surgical treatment of acetabular fractures.

We acknowledge that our study has several limitations, such as the great heterogeneity between the included studies, especially regarding rehabilitation protocols and the direct lack of correlation between these, the quality of reduction, and complications. Moreover, we included studies with patients with associated skeletal or non-skeletal injuries, which can be seen as a major bias as this can affect the choice and timing of rehabilitation protocols. However, our study aimed to systematically review the available literature on rehabilitation practices, including weight-bearing protocols, over time in surgically treated acetabular fracture patients and correlate these practices with functional outcomes. In this context, as previously stated, our findings corroborated other authors who analyzed permissive weight-bearing regimes for patients operated on for articular injuries of the lower limbs. Nevertheless, it is important to mention that all studies included in the final analysis were individual cohort studies or case-control studies with moderate-to-limited levels of evidence. This reinforces the need for urgent, robust, evidenced-based, randomized clinical trials to evaluate the benefits, risks, and indications of permissive weight-bearing after fixation of acetabular fractures. Finally, the use of the current existing patient-reported outcome measurements (PROMs) appears to be insufficient to truly assess the role of the “ability to walk” in terms of pain management, return to a pre-injury functional level, and quality of life, all of which are important components for better understanding the relationship between patient satisfaction and patient trust in the attending physician and the treatment carried out [58,59,60,61].

## 5. Conclusions

Our findings suggest that an accelerated postoperative rehabilitation protocol, including early permissive weight-bearing, does not appear to increase the risk of loss of reduction or the rate of complications after surgical treatment of acetabular fractures. However, a proper meta-analysis was not possible, and the heterogeneity of the included studies did not allow us to conclude anything about the potential biomechanical and clinical benefits nor the negative effects related to this rehabilitation regimen in terms of functional results. It is obvious that there is an inconsistent use of PROMs that allows objectively calculating the effect size of the accelerated protocol compared to restricted weight-bearing regimes. In this scenario, we pose the need for higher-level evidence to prove our hypothesis.

## Figures and Tables

**Table 1:** Characteristics of the 22 included studies.

Author(s)	Journal	Year Published	Grade of Recommendation/Level of Evidence	Methodological Index for Non-Randomized Studies (MINORS) Criteria
Fan, S. et al. [19]	Orthop Surg	2023	B/2b (individual cohort study or low-quality randomized control studies)	12 (non-comparative study)
Kojima, K.E. et al. [25]	Acta Ortop Bras	2022	C/4 (case series, low-quality cohort, or case-control studies)	20 (comparative study)
Yang, Y. et al. [36]	Orthop Surg	2022	C/4 (case series, low-quality cohort, or case-control studies)	12 (non-comparative study)
Patil, A. et al. [32]	Strategies Trauma Limb Reconstr	2021	C/4 (case series, low-quality cohort, or case-control studies)	12 (non-comparative study)
Li, Z. et al. [28]	Int Orthop	2021	C/4 (case series, low-quality cohort, or case-control studies)	21 (comparative study)
Selek, O. et al. [34]	HIP International	2019	C/4 (case series, low-quality cohort, or case-control studies)	13 (non-comparative study)
Chen, K. et al. [15]	J Orthop Trauma	2018	C/4 (case series, low-quality cohort, or case-control studies)	12 (non-comparative study)
Hue, A.G. et al. [22]	Orthop Traumatol Surg Res	2018	C/4 (case series, low-quality cohort, or case-control studies)	10 (non-comparative study)
Fahmy, M. et al. [18]	Injury	2018	B/2b (individual cohort study or low-quality randomized control studies)	15 (non-comparative study)
Kizkapan, T.B. et al. [24]	Acta Orthop Belg	2018	C/4 (case series, low-quality cohort, or case-control studies)	19 (comparative study)
Karin, M.A. et al. [23]	Injury	2017	C/4 (case series, low-quality cohort, or case-control studies)	13 (non-comparative study)
Gupta, S. et al. [20]	Chin J Traumatol	2017	C/4 (case series, low-quality cohort, or case-control studies)	11 (non-comparative study)
Hammad, A.S. et al. [21]	Injury	2017	B/2b (individual cohort study or low-quality randomized control studies)	13 (non-comparative study)
Park, K.S. et al. [31]	Injury	2017	C/4 (case series, low-quality cohort, or case-control studies)	13 (non-comparative study)
Elmadağ, M. et al. [17]	Orthopedics	2016	C/4 (case series, low-quality cohort, or case-control studies)	15 (non-comparative study)
Li, Y.L. and Tang, Y.Y. [27]	Injury	2014	C/4 (case series, low-quality cohort, or case-control studies)	15 (non-comparative study)
Magu, N.K. et al. [29]	J Orthop Traumatol	2014	C/4 (case series, low-quality cohort, or case-control studies)	13 (non-comparative study)
Elmadağ, M. et al. [16]	Orthop Traumatol Surg Res	2014	C/4 (case series, low-quality cohort, or case-control studies)	18 (comparative study)
Maini, L. et al. [30]	J Orthop Surg	2014	C/4 (case series, low-quality cohort, or case-control studies)	9 (non-comparative study)
Schwabe, P. et al. [33]	J Orthop Trauma	2014	C/4 (case series, low-quality cohort, or case-control studies)	15 (non-comparative study)
Li, H. et al. [26]	Injury	2013	C/4 (case series, low-quality cohort, or case-control studies)	13 (non-comparative study)
Uchida, K. et al. [35]	Eur J Orthop Surg Traumatol	2013	C/4 (case series, low-quality cohort, or case-control studies)	15 (non-comparative study)

Source: authors, 2024. Legend: Orthop Surg = *Orthopaedic Surgery*; Acta Ortop Bras = *Acta Ortopedica Brasileira*; Strategies Trauma Limb Reconstr = *Strategies in Trauma and Limb Reconstruction*; Int Orthop = *International Orthopaedics*; J Orthop Trauma = *Journal of Orthopaedic Trauma*; Orthop Traumatol Surg Res = *Orthopaedic Traumatology Surgery and Research*; Acta Orthop Belg = *Acta Ortopedica Belgica*; Chin J Traumatol = *Chinese Journal of Orthopaedics*; J Orthop Traumatol = *Journal of Orthopaedics and Traumatology*; J Orthop Surg = *Journal of Orthopaedic Surgery*; Eur J Orthop Surg Traumatol = *European Journal of Orthopaedic Surgery and Traumatology*.

**Table 2:** Sample demographics.

Parameter		
Male:female ratio *	721:207 (77.6%:22.3%)	*p* = 0.0005
Average age ± SD **	42.8 ± 10.9 years	
Letournel classification ***	401 elementary fractures 614 associated fractures	*p* = 0.21
Associated injuries	236 skeletal injuries 32 non-skeletal (excluding neurological) injuries 11 peripheral nerve injuries 8 non-specified multiple traumas	*p* = 0.09

Source: authors, 2024. Legend: SD = standard deviation. * 20 studies (929 patients). ** 21 studies. *** Three fractures considered non-classifiable, and one fracture classified as an isolated quadrilateral plate.

**Table 3:** Perioperative parameters and form of treatment.

Author(s)	Time to Surgery Mean ± SD (days)	Surgical Approach	Surgical Time Mean ± SD (minutes)	Blood Loss Mean ± SD (mL)
Fan, S. et al. [19]	8.7 ± 2.6 (range: 5–21)	Lateral-rectus	75 ± 29 (range: 35–150)	440 ± 153 (range: 250–1400)
Kojima, K.E. et al. [25]	N/A	N/A	N/A	N/A
Yang, Y. et al. [36]	7.1	Kocher–Langenbeck	135.8 (range: 90–230)	405.4 (range: 200–650)
Patil, A. et al. [32]	2.8	Kocher–Langenbeck (*n* = 15), iliofemoral (*n* = 1), or modified anterior intrapelvic approach (*n* = 7)	N/A	N/A
Li, Z. et al. [28]	N/A	Kocher–Langenbeck (*n* = 35) or N/A (*n* = 48)	154.97 ± 17.00	334.59 ± 23.73
Selek, O. et al. [34]	N/A	N/A	N/A	N/A
Chen, K. et al. [15]	9.2 ± 4.9 (range: 4–21)	Single modified ilioilioinguinal	182 ± 40	793 ± 228 (range: 500–1500)
Hue, A.G. et al. [22]	12 (range: 8–17)	Extended iliofemoral	240 (range: 180–360)	N/A
Fahmy, M. et al. [18]	8 ± 3 (range: 2–17)	Kocher–Langenbeck	N/A	range: 500–1000
Kizkapan, T.B. et al. [24]	2.3 (range: 1–6)	Kocher–Langenbeck	N/A	N/A
Karin, M.A. et al. [23]	5.4 (range: 1–18)	Ilioinguinal (*n* = 36), modified Stoppa (*n* = 4), and additional Kocher–Langenbeck (*n* = 7)	148.5 ± 33.8	741.2 ± 203.8
Gupta, S. et al. [20]	4.6 (range: 1–26)	Kocher–Langenbeck with trochanteric flip osteotomy	N/A	N/A
Hammad, A.S. et al. [21]	6.5	Kocher–Langenbeck	N/A	N/A
Park, K.S. et al. [31]	5.7 (range: 3–15)	Kocher–Langenbeck and additional mini iliofemoral	160 (range: 75–320)	N/A
Elmadağ, M. et al. [17]	N/A	Modified Stoppa	N/A	970 (range: 800–1250)
Li, Y.L. and Tang, Y.Y. [27]	6.6 (range: 2–15)	Ilioinguinal (*n* = 14), Kocher–Langenbeck (*n* = 11), and Ilioinguinal + Kocher–Langenbeck (*n* = 27)	N/A	N/A
Magu, N.K. et al. [29]	N/A	Kocher–Langenbeck with additional digastric trochanteric flip osteotomy (*n* = 3)	N/A	N/A
Elmadağ, M. et al. [16]	3.7	Ilioinguinal (*n* = 19) and modified Stoppa (*n* = 17)	N/A	1140 (range: 450–2150)
Maini, L. et al. [30]	Within 3 weeks of injury	Kocher–Langenbeck with digastric trochanteric flip osteotomy	150 (range: 90–240)	800 (range: 350–1800)
Schwabe, P. et al. [33]	N/A	Percutaneous	N/A	N/A
Li, H. et al. [26]	7.2 (range: 0–14)	Kocher–Langenbeck	120 (range: 105–180)	246 (range: 150–450)
Uchida, K. et al. [35]	10 (range: 1–32)	Ilioinguinal (*n* = 19), Kocher–Langenbeck (*n* = 33), ilioinguinal + Kocher–Langenbeck (*n* = 13), and others (Smith-Peterson (2) and iliofemoral (4)) (*n* = 6)	N/A	N/A

Source: authors, 2024. Legends: N/A = not available; mL = milliliters.

**Table 4:** Quality of reduction, postoperative rehabilitation protocol, and outcome measurement.

Author(s)	Quality of Reduction	Postoperative Rehabilitation Protocol	Outcome Measurement
Fan, S. et al. [19]	Excellent in 131 cases, good in 31 cases, and poor in 16 cases	Isometric contraction training of lower limb muscles carried out 24 h after operation, toe-touch weight-bearing permitted 6–10 weeks after surgery, and full weight-bearing depending on the patient’s general condition and fracture healing state.	Excellent in 125 cases, good in 26 cases, and fair in 27 cases (MDPS)
Kojima, K.E. et al. [25]	Satisfactory in 61 cases in the non-weight-bearing group and 59 cases in the immediate weight-bearing group	71 patients underwent rehabilitation with a non-weight-bearing protocol, while 66 patients underwent rehabilitation with immediate weight-bearing as tolerated.	N/A
Yang, Y. et al. [36]	Anatomic in 17 cases, imperfect in 3 cases, and poor in 4 cases	Physical therapy with isometric quadriceps- and abductor-strengthening exercises on the first postoperative day, passive hip movement at 2–3 days postoperatively, and active hip movement without weight-bearing at 3–4 weeks postoperatively. Patients with traumatic posterior hip dislocation maintained skeletal traction for 2–4 weeks before hip functional exercise. Partial weight-bearing gradually initiated at 8–12 weeks according to fracture healing.	Excellent in 10 cases, good in 6 cases, fair in 5 cases, and poor in 3 cases (MDPS)
Patil, A. et al. [32]	Acceptable in 23 cases	Patients were kept in bed for 2 weeks, followed by non-weight-bearing mobilization with the help of a walker for another 2 weeks. Partial weight-bearing was started at 1 month, which was increased to full weight-bearing at 4 months.	Mean modified MDPS of 14.95 (±3.46) and average HHS of 85.48 (±2.97)
Li, Z. et al. [28]	Excellent in 38 cases, good in 25 cases, fair in 17 cases, and poor in 3 cases	Isometric contraction training of the lower limbs was allowed right after the patient awoke from anesthesia. All patients remained non-weight-bearing for four weeks, and progressive weight-bearing was allowed after radiological evidence of fracture healing.	Excellent in 26 cases, good in 36 cases, fair in 13 cases, and poor in 8 cases (MDPS)
Selek, O. et al. [34]	Excellent in 20 cases, good in 24 cases, fair in 6 cases, and poor in 5 cases	Passive ROM exercises of the hip, including isotonic and isometric strengthening exercises applied just after the operation, and toe-touch weight-bearing from 6 to 12 weeks.	Excellent in 16 cases, good in 26 cases, fair in 10 cases, and poor in 3 cases (MDPS)
Chen, K. et al. [15]	Excellent in 17 cases, good in 4 cases, and poor in 1 case	Non-weight-bearing exercises were performed in bed within 4 weeks postoperatively, and patients were allowed to walk with a pair of crutches 4–6 weeks after operation and with a single crutch 6–12 weeks after operation.	Excellent in 14 cases, good in 6 cases, and poor in 2 cases (MDPS)
Hue, A.G. et al. [22]	Anatomic in all cases	Strict bedrest with continuous transtibial traction for 6 weeks. Passive mobilization of the hip after day 10. Raise from bed with 2 forearm crutches at week 6, with progressive painless resumption of weight-bearing.	N/A
Fahmy, M. et al. [18]	Anatomic in 24 cases and imperfect in 6 cases	Early ROM exercises and non-weight-bearing regimen on the affected limb for 6 weeks, followed by partial weight-bearing until 12 weeks, finally progressing to full weight-bearing at 12 weeks.	Excellent to good in 26 patients and fair to poor in 4 patients (MDPS)
Kizkapan, T.B. et al. [24]	Excellent in 6 cases, good in 13 cases, fair in 2 cases, and poor in 5 cases	All patients were allowed partial weight-bearing 3 months postoperatively and started full weight-bearing at 4–6 months postoperatively.	Excellent in 6 cases, good in 15 cases, and fair in 5 cases (MDPS)
Karin, M.A. et al. [23]	Anatomic in 23 cases, imperfect in 9 cases, and poor in 3 cases	ROM started from postoperative day 1, with weight-bearing delayed until full radiological and clinical unions were evident.	Excellent in 13 cases, good in 23 cases, fair in 3 cases, and poor in 1 case (MDPS)
Gupta, S. et al. [20]	N/A	Patients allowed for sitting, side turning, and pelvic lifting exercises on postoperative day 1, with toe-touch weight-bearing allowed within the first week and full weight-bearing allowed at the end of 3 months.	Excellent in 16 cases, good in 6 cases, and fair in 2 cases (MDPS)
Hammad, A.S. et al. [21]	Anatomic in 21 cases, imperfect in 4 cases, and poor in 9 cases	Non-weight bearing for 4 weeks, protected weight-bearing for 8 weeks, and full-weight bearing after 12 weeks.	Excellent to good in 25 cases and fair to poor in 9 cases (MDPS)
Park, K.S. et al. [31]	Anatomic in 12 cases, imperfect in 6 cases, and poor in 5 cases	Active ROM started the day after surgery, non-weight-bearing walking with two crutches from postoperative day 3, partial weight-bearing at 6 weeks, and full weight-bearing at 12 weeks.	Excellent in 15 cases, good in 5 cases, fair in 1 case, and poor in 2 cases (HHS)
Elmadağ, M. et al. [17]	Anatomic in 29 cases, imperfect in 5 cases, and poor in 2 cases	Flat-footed weight-bearing for 12 weeks.	Excellent in 14 cases, good in 12 cases, fair in 5 cases, and poor in 5 cases (HHS); excellent in 13 cases, good in 20 cases, fair in 2 cases, and poor in 1 case (MDPS)
Li, Y.L. and Tang, Y.Y. [27]	Excellent in 22 cases, good in 15 cases, fair in 6 cases, and poor in 9 cases	Sit up in bed on the first postoperative day with active and passive functional exercises on the operated hip and progressive resistance exercises of the hip adductors, quadriceps, and hamstrings. Patients encouraged to use walkers between 1 and 6 weeks and crutches between 6 and 12 weeks. Full weight-bearing according to tolerance after 12 weeks.	Excellent in 24 cases, good in 19 cases, fair in 2 cases, and poor in 7 cases (HHS); excellent in 14 cases, good in 29 cases, fair in 2 cases, and poor in 7 cases (MDPS)
Magu, N.K. et al. [29]	Excellent in 10 cases, good in 8 cases, fair in 5 cases, and poor in 3 cases	Intermittent, pain-free quadriceps, hip, and knee flexion exercises with traction starting on the second postoperative day, partial weight-bearing permitted 6 weeks after surgery, and gradually progressing to full weight-bearing at 12 weeks.	Excellent in 14 cases, good in 6 cases, fair in 3 cases, and poor in 3 cases (MDPS)
Elmadağ, M. et al. [16]	N/A	Crutches used for 6 weeks with weight-bearing not permitted, followed by one crutch for 6 more weeks, with partial weight-bearing allowed. Active and passive ROM exercises started in the early postoperative period.	Excellent in 21 cases, good in 12 cases, and fair in 3 cases (HHS); excellent in 18 cases, good in 14 cases, and fair in 4 cases (MDPS)
Maini, L. et al. [30]	Anatomic in 6 cases and satisfactory in 16 cases	Skeletal traction for 3 weeks, non-weight-bearing status for 6–12 weeks, depending on stability and fixation of the joint, and full weight-bearing after 12–20 weeks.	Extremely good in 6 cases, good in 13 cases, medium in 2 cases, and fair in 1 case
Schwabe, P. et al. [33]	Anatomic in all cases	Supervised mobilization with 30 kg of weight-bearing on the ipsilateral extremity with crutches or a mobile walking device started during the first day after the operation, with full weight-bearing after 6 weeks postoperatively.	Excellent in 8 patients and good in 4 patients (HHS)
Li, H. et al. [26]	Excellent in 45 cases, good in 10 cases, and fair in 2 cases	Joint exercise recommended as tolerated by pain, activities limited for an average of 12 weeks before partial weight-bearing was permitted, depending on the fracture stability, and full weight-bearing only after confirmed clinical and radiological fracture union.	Excellent or extremely good in 45 cases, good in 8 cases, fair in 2 cases, and poor in 2 cases
Uchida, K. et al. [35]	Anatomic in 42 cases, satisfactory in 27 cases, and unsatisfactory in 2 cases	Patients enrolled in physical therapy program on the third postoperative day, starting with hip (affected side) abduction and flexion, followed by isometric and then isotonic exercise, allowing sitting from 1 week and walking using a single cane without orthosis from 10 weeks.	N/A

Source: authors 2024. Legends: N/A = not available; ROM = range of motion; MDPS = Merle D’Aubigné and Postel score; HHS = Harris hip score.

**Table 5:** Follow-up period and complications.

Parameter		
Follow-up	From 6 weeks to 9 years	
Complication(s)	None in 12 cases	
	Heterotopic ossification in 52 cases	5.1%
	Posttraumatic hip arthritis in 41 cases	4.0%
	AVN of femoral head in 17 cases	1.6%
	Thromboembolic complications in 24 cases	2.3%
	Postoperative peripheral nerve injuries in 28 cases	2.7%
	Sciatic nerve palsy in 14 cases	
	Lateral femoral cutaneous nerve palsy in 13 cases	
	Obturator nerve palsy in 1 case	
	Others	4.1%
	Partial iliac vein damage in 1 case	
	Massive bleeding in 3 cases	
	Persistent drainage in 5 cases	
	Wound infection in 13 cases	
	Incisional hernia with mild symptoms 1 year after surgery in 1 case	
	Implant loosening or irritation in 4 cases	
	Loss of reduction in 12 cases	
	Delayed union in 2 cases	
	Femoroacetabular pincer-type impingement in 1 case	

Source: authors, 2024.

## Data Availability

The original contributions presented in the study are included in the article, further inquiries can be directed to the corresponding author/s.
